# Application of Cornelian Cherry Iridoid-Polyphenolic Fraction and Loganic Acid to Reduce Intraocular Pressure

**DOI:** 10.1155/2015/939402

**Published:** 2015-06-01

**Authors:** Dorota Szumny, Tomasz Sozański, Alicja Z. Kucharska, Wojciech Dziewiszek, Narcyz Piórecki, Jan Magdalan, Ewa Chlebda-Sieragowska, Robert Kupczynski, Adam Szeląg, Antoni Szumny

**Affiliations:** ^1^Department of Pharmacology, Wrocław Medical University, 50-345 Wrocław, Poland; ^2^Ophthalmology Clinic, Uniwersytecki Szpital Kliniczny, 50-556 Wrocław, Poland; ^3^Department of Fruit and Vegetables Technology, Wrocław University of Environmental and Life Sciences, 51-630 Wrocław, Poland; ^4^Arboretum and Institute of Physiography in Bolestraszyce, 37-722 Bolestraszyce, Poland; ^5^Department of Turism & Recreation, University of Rzeszow, 35-959 Rzeszów, Poland; ^6^Department of Environment Hygiene and Animal Welfare, Wrocław University of Environmental and Life Sciences, 51-630 Wrocław, Poland; ^7^Department of Chemistry, Wrocław University of Environmental and Life Science, 50-375 Wrocław, Poland

## Abstract

One of the most common diseases of old age in modern societies is glaucoma. It is strongly connected with increased intraocular pressure (IOP) and could permanently damage vision in the affected eye. As there are only a limited number of chemical compounds that can decrease IOP as well as blood flow in eye vessels, the up-to-date investigation of new molecules is important. The chemical composition of the dried Cornelian cherry (*Cornus mas* L.) polar, *iridoid-polyphenol*-rich fraction was investigated. Loganic acid (50%) and pelargonidin-3-galactoside (7%) were found as the main components. Among the other constituents, iridoid compound cornuside and the anthocyans cyanidin 3-*O*-galactoside, cyanidin 3-*O*-robinobioside, and pelargonidin 3-*O*-robinobioside were quantified in the fraction. In an animal model (New Zealand rabbits), the influence of loganic acid and the polyphenolic fraction isolated from Cornelian cherry fruit was investigated. We found a strong IOP-hypotensive effect for a 0.7% solution of loganic acid, which could be compared with the widely ophthalmologically used timolol. About a 25% decrease in IOP was observed within the first 3 hours of use.

## 1. Introduction

The term “glaucoma” involves a range of diseases associated with progressive optic nerve damage, including typical morphological changes of its disk and characteristic loss of the field of vision, which are often accompanied by increased intraocular pressure (IOP) [1]. Risk factors of glaucoma-associated optic nerve damage include increased IOP, positive family history, systemic arterial hypotension or hypertension, and ischaemia-aggravating diseases such as diabetes [2, 3].

The maintenance of IOP at a desired level is particularly important in the therapy of glaucoma [4]. Results of published studies demonstrate that a reduction in IOP is associated with a lower risk of glaucoma progression, owing to the less rapid optic nerve damage and reduced loss of the visual field [5–7]. Pressure reduction offered by the currently used medication is based on reduced production or increased outflow of aqueous media [8], some of which also have neuroprotective properties or are able to improve vascular flow. Nowadays, the following groups of medication are used for glaucoma treatment: *β*-adrenergic receptor antagonists, prostaglandin analogues, *α*_2_-adrenergic receptor agonists, carbonic anhydrase inhibitors, cholinergic agents, and hyperosmotic drugs [9, 10].

Only a few studies have been aimed at the effect of compounds of natural origin on the IOP, as well as other glaucoma-progressing factors, such as vascular blood flow, antioxidative effects, or neuroprotection. The majority of these studies were conducted* in vitro*, but a few were* in vivo* and reached clinical trials. The difficulties encountered in the evaluation of natural compounds on the progress of glaucoma-associated neuropathy are a consequence of long-term disease development but are also because of the limited possibility to use proper animal models [11, 12].

One of several plant-origin compounds with a proven IOP-reducing effect is forskolin, which is obtained from* Coleus forskohlii* (Lamiaceae). Following intraconjunctival administration, the compound stimulates adenylate cyclase, which leads to the activation of cyclic adenosine monophosphate in ciliary epithelium and the reduction of IOP, resulting from reduced production of aqueous products in rabbits, monkeys, and humans [13, 14]. Similarly, natural bromophenols isolated from red algae belonging to the Rhodomelaceae family, such as vidalol B, present activity as carbonic anhydrase inhibitors and may become a valuable IOP-reducing drug in the future [15]. Recently, marijuana was considered as an IOP-reducing natural compound. However, because of a high ratio of nonresponders, short half-life, and considerable toxicity, the substance was not qualified for medicinal use [16–18]. Among other plants,* Ginkgo biloba* leaf extracts may be used for adjunct glaucoma therapy. The extract contains polyphenolic flavonoid compounds, as well as terpenoids, and is able to scavenge free radicals at the mitochondrial level in order to improve blood flow. In addition, the substance prevents neurotoxicity of glutamate, which may be important for a neuroprotective effect, and inhibits lipid peroxidation [19, 20]. Although it does not affect the IOP, it may improve vascular blood flow into the optic nerve [21]. Clinical trials have indicated that anthocyanins and* Ginkgo biloba* extract may be effective in improving the visual function and the visual field of patients with normal-tension glaucoma [20, 22]. Nowadays, the only drug of natural origin currently used in glaucoma treatment is pilocarpine, which is the alkaloid from Jaborandi (*Pilocarpus microphyllus*) leaves. Its pharmacological activities are connected with properties of a muscarinic agonist with no nicotinic effects [23, 24]. One of the most popular plant extracts traditionally used in eyes diseases in Europe, especially for the inflammation of eyes, is Eyebright (*Euphrasia*). Its most important constituents that affect the eyes are iridoid glycosides, including aucubin, geniposide, catalpol, and luproside [25–27]. Although there are no relevant clinical studies, an established traditional use of those eye drops proves their efficacy in conjunctivitis [28].

To the best of our knowledge, there is a lack of data regarding the effect of loganic acid or Cornelian cherry preparations on IOP or vascular flow in the eye. We have not found any loganic-acid-containing or Cornelian-cherry-based preparations registered in the world, which could be used for the therapy of glaucoma, reduction of IOP, or increasing vascular blood flow in the eye.

The aim of our study was to investigate the influence of* Cornus mas* iridoid-rich extract as well as its predominant compound, loganic acid, on IOP.

## 2. Materials and Methods

### 2.1. Evaluation of Cornelian Cherry Constituents

Raciborski-variety Cornelian cherries (*Cornus mas* L.) were obtained from the Bolestraszyce Arboretum and Institute of Physiography, Poland, in September 2012. The plant material was authenticated by Professor Jakub Dolatowski, and the voucher specimen (12402) was deposited at the Herbarium of Arboretum and Institute of Physiography in Bolestraszyce, Poland. The polyphenolic and loganic acid iridoid fractions were prepared in the Department of Fruit, Vegetable and Cereals Technology at Wroclaw University of Environmental and Life Science, according to the procedure described in our previous publication [29] and patent [29]. Ripe Cornelian cherries were washed, frozen, and stored at −20°C until processing. After freezing, removing stones, defrosting, and depectinisation, we obtained the juice. The juice was purified on an Amberlite XAD-16 resin column. Iridoids and phenolics were eluted with 80% ethanol. The eluent was concentrated under vacuum at 40°C and lyophilised. About 20 g of lyophilisate from the iridoid-polyphenolic fraction was obtained from 5 kg of fruit. After freeze drying, the samples were ground into a powder using a laboratory mill. The structure of the compounds in the iridoid-phenolic fraction was determined by UPLC-MS analysis.

### 2.2. Isolation of Anthocyan-Irdoid Fraction and Pure Loganic Acid

The easy, effective, and low-cost approach for isolation of loganic acid as well as the anthocyanin fraction was developed in our research group [30]. In brief, freeze-died Cornelian fruit powder (as described above) was dissolved in 30% ethanol and separated using column chromatography (polyamide). Elution with 50% ethanol (and 1% acetic acid) afforded the anthocyanin fraction and pure loganic acid. Applying the described procedure to 1 kg of fresh fruit allowed us to obtain 4.0 g of the anthocyanin fraction and 1.5 g of pure loganic acid.

The structure and purity of loganic acid was determined by NMR and UPLC-MS analyses.

### 2.3. Identification of Compounds by UPLC-MS/MS and HPLC

Compounds of the phenolic-iridoid and loganic acid fractions were identified by applying the method described by Kucharska et al. [30], using the acuity ultra-performance liquid chromatography (UPLC) system coupled with a quadrupole time-of-flight (Q-TOF) MS instrument (Waters Corp., Milford, MA, USA) with an electrospray ionisation (ESI) source. Separation was achieved on the Acquity BEH C18 column (100 mm × 2.1 mm, i.d. 1.7 mm; Waters). Detection wavelengths were set to 245 and 520 nm. The mobile phase was a mixture of 4.5% formic acid (A) and acetonitrile (B). The gradient program was as follows: initial conditions: 99% (A), 12 min; 75% (A), 12.5 min; 100% (B), 13.5 min; 99% (A). The flow rate was 0.45 mL/min and the injection volume was 5 *μ*L. The column was operated at 30°C. The instrument was operated in both positive- (anthocyanins) and negative-ion (iridoids) modes, scanning the* m/z* from 100 to 1500 at a scan rate of 2.0 s/cycle. The major operating parameters for the Q-TOF MS were set as follows: capillary voltage 2.0 kV, cone voltage 45 V, cone gas flow of 11 L/h, collision energy 28 eV, source temperature 100°C, desolvation temperature 250°C, collision gas argon, and desolvation gas (nitrogen) flow rate 600 L/h. The data were collected with Mass-Lynx V 4.1 software.

Additionally, compounds in the obtained phenolic-iridoid and loganic acid fractions were quantified using the Dionex HPLC system (Germering, Germany) equipped with the Ultimate 3000 model diode-array detector. The detector cooperated with the LPG-3400A pump, EWPS-3000SI autosampler, column thermostat TCC-3000SD, and Chromeleon version 6.8 software. A Cadenza Imtakt C5–C18 column (75 × 4.6 mm) was used with a guard column. The mobile phase was composed of solvent A (4.5% formic acid, v/v) and solvent B (acetonitrile). The elution system was as follows: 0-1 min, 5% B; 20 min, 25% B; 21 min, 100% B; 26 min, 100% B; 27 min, 5% B. The flow rate of the mobile phase was 1.0 mL/min and the injection volume was 20 *μ*L. The column was operated at 30°C. The runs were monitored at wavelengths of 245 (iridoids) and 520 nm (anthocyanins). Iridoids were quantified as loganic acid and anthocyanins as cyanidin 3-*O*-glucoside. The results were calculated as milligrams of compound in 1 g dry weight of Cornelian cherry powder (mg/g d.w.). All determinations were performed in triplicate.

### 2.4. NMR Analysis

The NMR spectrum of loganic acid was measured on an NMR Bruker AVANCE 600 MHz spectrometer in the Laboratory of Structural Analysis at the Technical University of Wrocław. The chemical structure was established on the basis of ^1^H and ^13^C NMR as well as correlative HSQC, HMBC, and ROESY spectra. The measurement was performed at 24°C.

### 2.5. Reference Material

The maleate salt of timolol (1% conc. eye-drops) was purchased as commercially available Oftan Timolol (Santen, Poland) and used as in the reference.

### 2.6. Biological Assays

For the evaluation of the effect of loganic acid or the polyphenolic fraction on IOP and blood flow in blood vessels of the iris, 14 young, sexually mature, New Zealand white rabbits, aged between 6 and 12 months, were used: 7 males and 7 females. During the experiment, the rabbits were kept in individual cages at 21–23°C. The animals were fed with a full-meal blend rabbit feed (special blend for the time of biological testing LSK, Agropol S.J.)* ad libitum* with unlimited access to drinking water.

Before the experiment itself, the animals were kept under 4-week quarantine. The behaviour of the animals and their physical condition were observed during that time. The measured data, throughout all experiments, were obtained by one person in order to achieve domestication and to reduce the amount of stress, which could interfere with results of the experiment.

A 0.7% solution of loganic acid or the polyphenolic fraction was administered with an aqueous solution of artificial tears containing 0.15% sodium hyaluronate (vehicle).

In the first part of the study, each animal was given 0.7% loganic acid or polyphenolic fraction by intraconjunctival administration of one drop, corresponding to a volume of 50 *µ*L, to the right eye (study group), whereas the vehicle in one drop (50 *µ*L) was administered to the left eye as a placebo (control group). Then, the IOP was measured in both eyes at six time points: before administration and 1, 2, 3, 4, and 5 hours after administration of loganic acid and the vehicle. Induction tonometer Icare, requiring no prior anaesthesia, was used for the measurement of the IOP. Reproducibility of the data has widely been described by other authors [31–34].

In the second part of the study, each animal was given 0.7% loganic acid through intraconjunctival administration in one drop (50 *µ*L) to the right eye, whereas the vehicle was again administrated to the left eye in one drop (50 *µ*L). Blood flow in the iris was measured before administration and 1, 2, 3, 4, and 5 hours after administration. The measurement was completed using a laser Doppler flow meter (Laser Blood Flow Monitor MBD_3_, Moor Instruments, GB). Following local anaesthesia of the cornea (Alcaine, Alcon), the laser was placed in direct contact with the cornea (ca. 2 mm from the corneal limbus). The laser beam was directed perpendicularly to the surface of the iris. The measurement time was 50 seconds. During that time, the apparatus performed 500 measurements of momentary capillary flow.

The interval between the first and the second parts of the described experiments was 2 days.

### 2.7. Statistical Analysis

The following tests were used to determine differences between groups: the nonparametric Wilcoxon test was used for comparison of IOP values and Student's* t*-test was used for comparison of iris blood flow values.

## 3. Results and Discussion

The easy, effective, and low-cost approach for the isolation of loganic acid, as well as the anthocyanin fraction, was developed in our research group and led to the isolation of biologically active compounds.

The chemical composition of the polar fraction obtained from Cornelian cherry fruit is presented in Table 1. The predominant compounds were iridoids, loganic acid, together with cornuside (about 55%) in the mixture. Of the anthocyanins, cyanidin and pelargonidin-3-galactoside prevailed. Among the other constituents, the iridoid compound cornuside and the following anthocyans cyanidin 3-*O*-galactoside, cyanidin 3-*O*-robinobioside, and pelargonidin 3-*O*-robinobioside were quantified in fraction.

Contrary to our results, the recent study by Pawlowska et al. [35] shows pelargonidin-3-glucoside together with cyanidin galactoside as the main flavonoids. The iridoid and flavonoid fraction of* C. mas* is quite different to the* C. officinalis* traditionally used in Chinese medicine to increase urine flow and decrease blood pressure [36]. The main constituents of the iridoid fraction of* C. mas *fruits were loganic acid and morroniside, which are considered as biologically active compounds [37].

**Figure 1:**
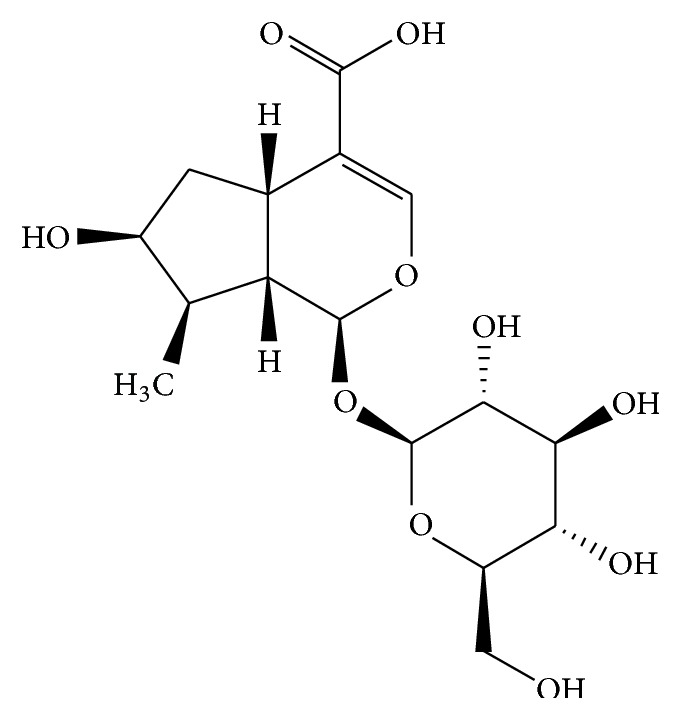
Structure of loganic acid.

The structure of loganic acid (Figure 1) was confirmed by NMR, obtaining appropriate chemical-shift values: ^13^C NMR (DMSO-D_6_) *δ* (ppm): 168.56 -CO_2_H; 150.55 C-3; 113.05 C-4; 98.97 C-1′; 96.48 C-1; 77.66 C-5′; 77.22 C-3′; 73.61 C-2′; 72.64 C-7; 70.54 C-4′; 61.60 C-6′; 45.20 C-9; 42.21 C-6; 40.97 C-8; 31.34 C-5; 14.01 C-10. ^1^H NMR (DMSO-D_6_) *δ* (ppm): 7.29 (1H, s, H-3); 5.09 (1H, d,* J* = 4.9 Hz H-1); 4.48 (1H, d,* J* = 7.9 Hz H-1′); 3.88 (1H, t,* J* = 4.7 Hz); 3.66 (1H, d,* J* = 11.7 Hz H-6′a); 3.44 (1H, dd* J* = 11.7 and 6.4 Hz H-6′b); 3.15 (1H, dd,* J* = 9.0 and 8.6 Hz); 3.14 (1H, m, H-5′); 3.05 (1H, dd,* J* = 9.4 and 9.0 Hz, H-4′); 2.97 (1H, t,* J* = 8.6 Hz, H-3′); 2.92 (1H, q,* J* = 7.9 Hz, H-5); 2.07 (1H, dd,* J* = 13.2 and 7.9 Hz, H-6); 1.81 (1H, ddd,* J* = 8.6, 7.9 and 4.9 Hz, H-9); 1.71 (1H, m, H-8); 1.44 (1H, ddd,* J* = 13.2, 7.9 and 4.7 Hz, H-6); 0.98 (3H, d,* J* = 6.8 Hz, CH_3_-). The chemical structure of the analysed compound was typical for iridoids and the diagnostic for interpretation signals was the ether enolic proton (C-3) at 7.29 ppm, with a carbon at 150.55 ppm correlated in the HMBC spectrum to acetal H-1 proton and carboxyl group present in the loganic acid structure. Additionally, a sugar moiety coupled to the iridoid was identified according to the available ^13^C shifts database in [38]. The obtained spectra (chemical shifts of protons and carbon atoms) were consistent with previously reported data [39, 40] and supported the results of the HPLC and MS ([M − H]^−^ ion at* m/z* 375) analyses. However, this is the first spectrum of loganic acid recorded in deuterated dimethyl sulfoxide.

The presence of loganic acid (Figure 1) and cornuside in* C. mas* fruits was also proven by Deng et al. [41], but on the basis of UV spectroscopy and the molecular mass. The same authors identified loganin (methyl ester of loganic acid) as well as sweroside, although they did not perform quantitative analyses. Loganic acid belongs to a large natural-compound group of terpenes called iridoids, which has also been found in plants like* Gentiana linearis* [42]*, Strychnos nux-vomica* [40] or, recently,* Cornus officinalis* fruits [37, 43], but in much lower concentrations. The related* C. mass *species,* C*.* officinalis*, grown in Asia has a strong beneficial effect on diabetic nephropathy [44]. Its activity was proven both in an animal model [45] and on the basis of traditional Chinese medicine [46]. According to Jiang, this activity is strongly connected with the methyl ester of loganic acid (loganin). The biological activity of loganic acid was recognised slightly, and its anti-inflammatory [47], antimicrobial [39], or antiproliferative [48] properties were proven in the presence of the other iridoids isolated from the corresponding plants.

**Figure 2:**
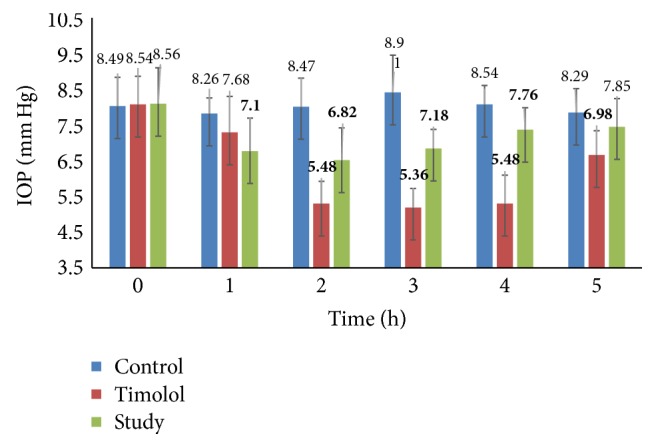
Mean values (mm Hg) of IOP after intraconjunctival administration of the anthocyan-irdoid fraction (study) and timolol. The data are expressed as the mean values, and the error bars represent standard deviations. Statistically significant values are shown in bold type.

**Figure 3:**
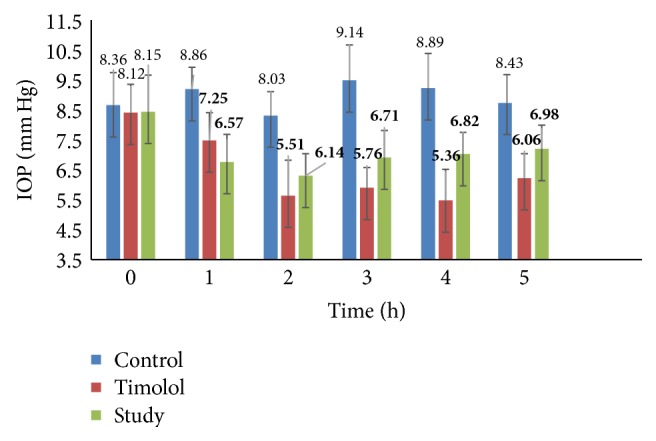
Mean values (mm Hg) of IOP after intraconjunctival administration of 0.7% loganic acid (study group) and timolol. The data are expressed as the mean values, and the error bars represent standard deviations. Statistically significant values are shown in bold type.

The effects of the anthocyan-irdoid fraction and pure loganic acid at a concentration of 0.7% on the IOP are presented in Figures 2 and 3.

The slight effect of the anthocyan-irdoid fraction was investigated. A 19% IOP decrease was measured 2-3 h after ocular administration. This effect was weakened after 4 h. During this assessment, timolol gave 35 and 39% decreases in IOP. The last values are in agreement with the results of other authors that observed similar IOP reduction in rat [49] or dog models [50].

More promising results were observed in the case of ocular administration of 0.7% loganic acid. A clear, statistically significant (*p* < 0.05) reduction in IOP 1, 2, 3, and 5 hours after administration of 0.7% loganic acid was found compared to the administration of the vehicle (Figures 2 and 3). An approximately 25% statistically significant reduction in IOP was observed during the first 3 hours. At the end of the experiment, 5 hours after initiating the experiment, a 15% decrease was measured. Although, compared to the loganic acid, timolol maleate gave a stronger effect on the IOP, it is our opinion that this effect may indicate the possibility of practical use.

One of several plant-origin compounds with proven IOP-reducing effect is forskolin, obtained from* Coleus forskohlii* (Lamiaceae). Following intraconjunctival administration, the compound stimulates adenylate cyclase, which leads to the activation of cyclic adenosine monophosphate in ciliary epithelium and a reduction in IOP, resulting from the reduced production of aqueous fluid in rabbits, monkeys, and humans [13, 14]. Nowadays, the only drug of natural origin currently used in glaucoma treatment is pilocarpine, the alkaloid from Jaborandi (*Pilocarpus microphyllus*) leaves. Its pharmacological activities are probably connected with its properties as a muscarinic agonist, with no nicotinic effects [23, 24].

**Figure 4:**
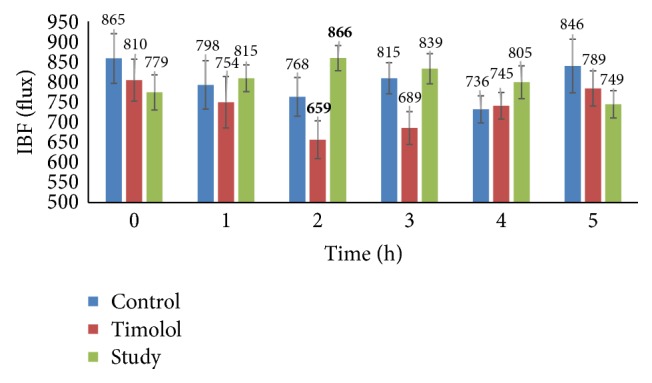
Mean of iris blood flow (flux) after intraconjunctival administration of timolol and loganic acid (study). The data are expressed as the mean values, and the error bars represent standard deviations. Statistically significant values are shown in bold type.

The second cause of glaucoma is reduced blood flow in the ciliary arteries. We measured the values of iris blood flow of the active compound, that is, loganic acid. The data are shown in Figure 4. The statistically significant (*p* < 0.05) increase in the mean iris blood flow was observed only 2 hours after the administration of 0.7% loganic acid.

No IOP-reducing effect was observed after oral administration of the Cornelian cherry fruit lyophilisate. The evaluation of possible allergic reactions in the tested eyes was also performed. During the experiment, the allergenic properties of the extract or loganic acid were evaluated using a visual method [51]. No irritant or allergenic effect of the preparation at the administration time or during the experiment was found.

Although there are great numbers of papers considering the effect of timolol as a hypotensive agent in eyes, the exact mechanism is still unclear [52, 53]. The proposed mechanism of reduction of IOP consists of reducing the secretion of aqueous humour in the ciliary body epithelium by inhibiting *β*-adrenergic receptors. According to other authors [54], loganic acid isolated from* Gentiana crassicaulis* possesses anti-inflammatory properties by inhibitory effects on LPS-induced nitric oxide (NO). Also, [55] shows that the production and increased concentration of NO in aqueous humour are connected with the pathogenesis of glaucoma. An elevated level of NO was observed in primary open-angle glaucoma patients [56]. We suspect that an NO-inhibitory effect of loganic acid could result in the observed IOP and the blood flow in the eye.

Very promising data obtained during the experiment could be the first step towards clinical assays. Although they were achieved in an animal (rabbit) model, according to our knowledge, they could be related to human eyes for glaucoma therapy. Also, this is the first ophthalmology research in which the active compound belongs to a subclass of terpenoids, that is, iridoids. Nowadays, there are no data about the ocular pharmacological activity of other iridoids, so other compounds from this group should be taken into consideration.

## 4. Conclusions

Intraconjunctival administration of loganic acid (0.7%) reduces the IOP in rabbit eyes. Our data, obtained in an animal model, directly suggest that loganic acid could be further used in the adjunct therapy of glaucoma in patients with increased IOP. It could also be beneficial for the intensification of vascular flows in diabetic and hypertensive retinopathy or other conditions of ocular blood vessels (e.g., venous thrombosis or arterial embolism).

## Supplementary Material



## Figures and Tables

**Table 1:** The chemical composition of the iridoid-polyphenolic fraction of Cornelian cherries (Raciborski variety).

Compound	[M − H]^−^/[M + H]^+^ (*m*/*z*)	Ionisation	(mg/g)	%
Iridoids				
**Loganic acid (LA)**	375.1276	−	216.69 ± 4.25	50.24
Cornuside (Co)	541.1566	−	21.55 ± 0.29	5.00
**Total**			**238.24**	

Anthocyanins				
Delphinidin 3-*O*-galactoside (Df-gal)	465.1034	+	0.63 ± 0.02	0.15
Cyanidin 3-*O*-galactoside (Cy-gal)	449.1063	+	15.79 ± 0.26	3.66
Cyanidin 3-*O*-robinobioside (Cy-rob)	595.1713	+	6.38 ± 0.04	1.48
Pelargonidin 3-*O*-galactoside (Pg-gal)	433.1125	+	29.94 ± 0.24	6.94
Pelargonidin 3-*O*-robinobioside (Pg-rob)	579.1766	+	5.95 ± 0.08	1.38
**Total**			**58.69**	

Flavonols				
Quercetin 3-*O*-glucuronide (Q-glucr)	477.0665	−	4.86 ± 0.17	1.13
Kaempferol 3-*O*-galactoside (Kf-gal)	447.0916	−	4.03 ± 0.20	0.93
**Total**			**8.89**	

Phenolic acids				
3-*O*-Caffeoylquinic acid (3-CQA)	353.0879	−	3.47 ± 0.08	0.81
5-*O*-Caffeoylquinic acid (5-CQA)	353.0879	−	10.89 ± 0.19	2.52
**Total**			**14.36**	
