# Supplementary material for: Application of Cornelian Cherry Iridoid-Polyphenolic Fraction and Loganic Acid to Reduce Intraocular Pressure
Source: Evid Based Complement Alternat Med. 2015 Jun 1;2015:939402. doi: 10.1155/2015/939402 (PMC4466386; doi:10.1155/2015/939402)
Supplement: Supplementary file 1 [file 939402.f1.zip › 939402supp1190361_v1.pdf]

1H spectrum of isolated loganic acid:

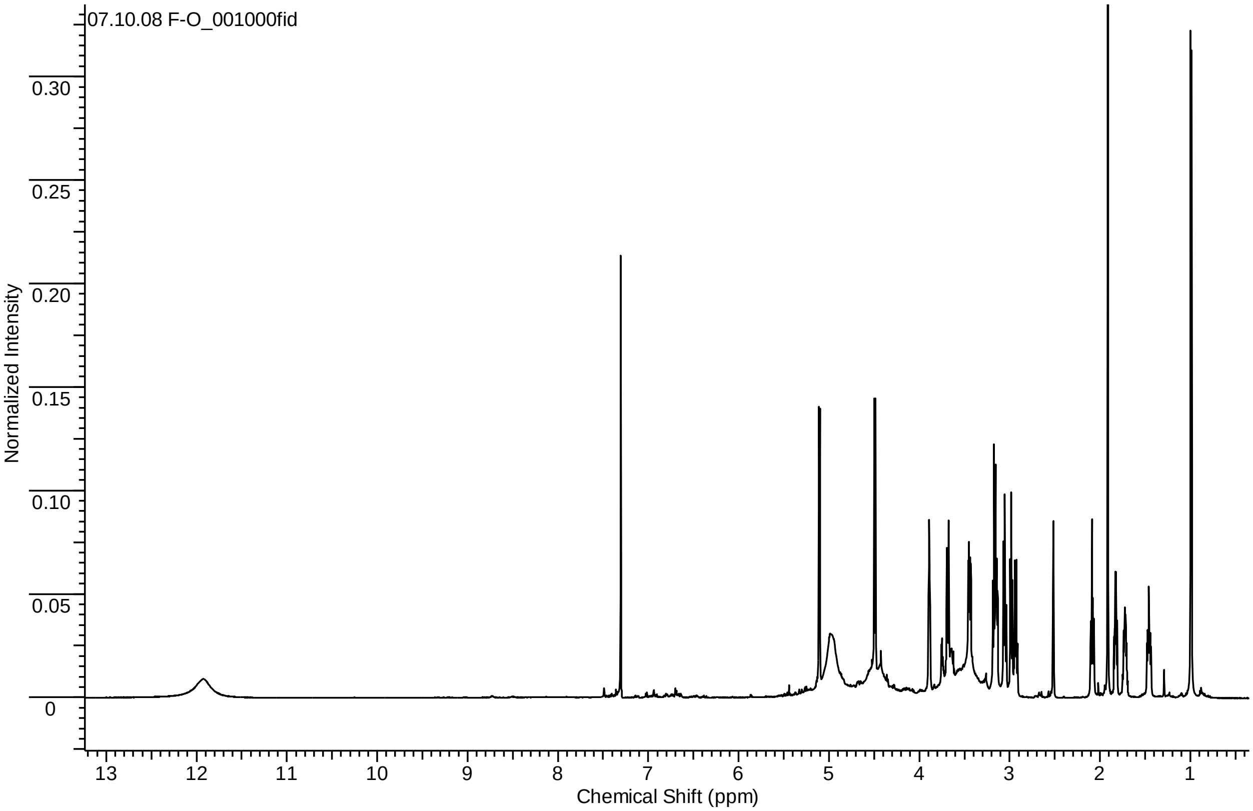

13C spectrum of loganic acid:

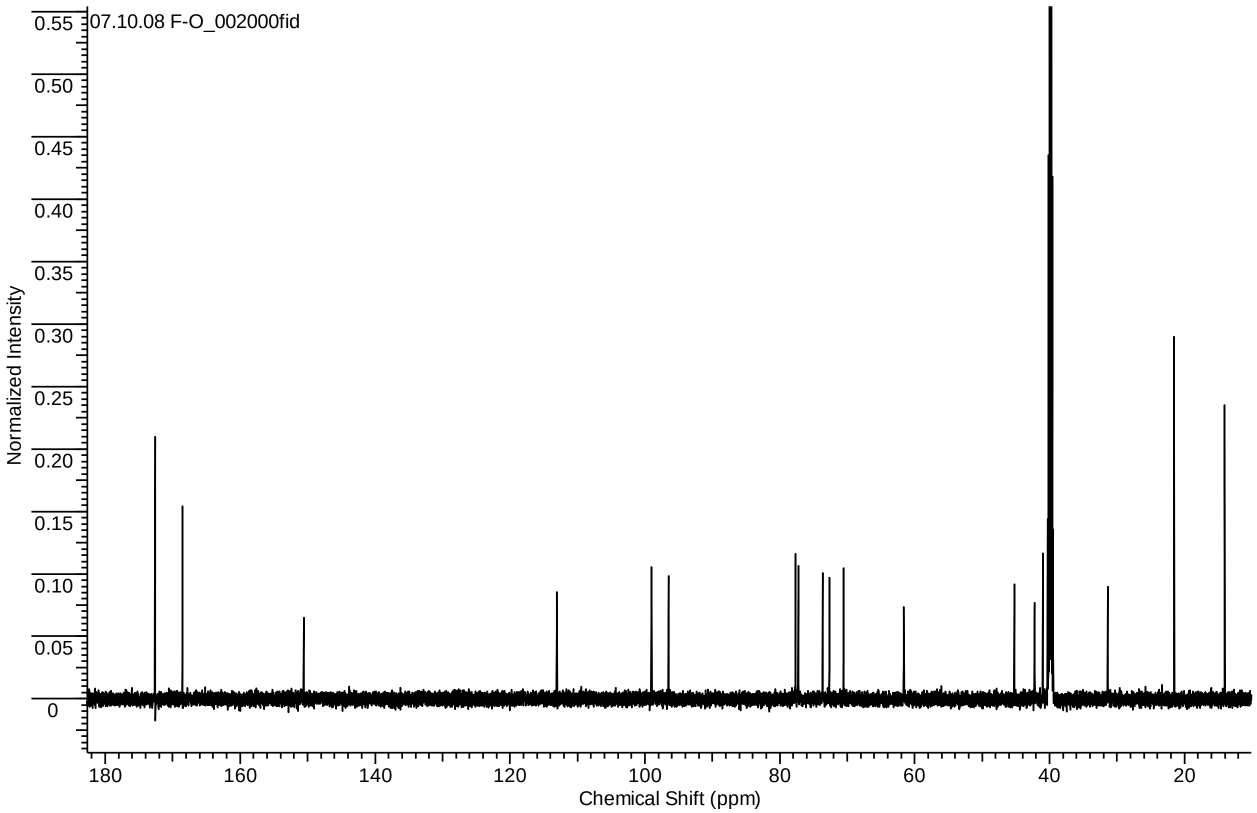

HSQC spectrum of loganic acid:

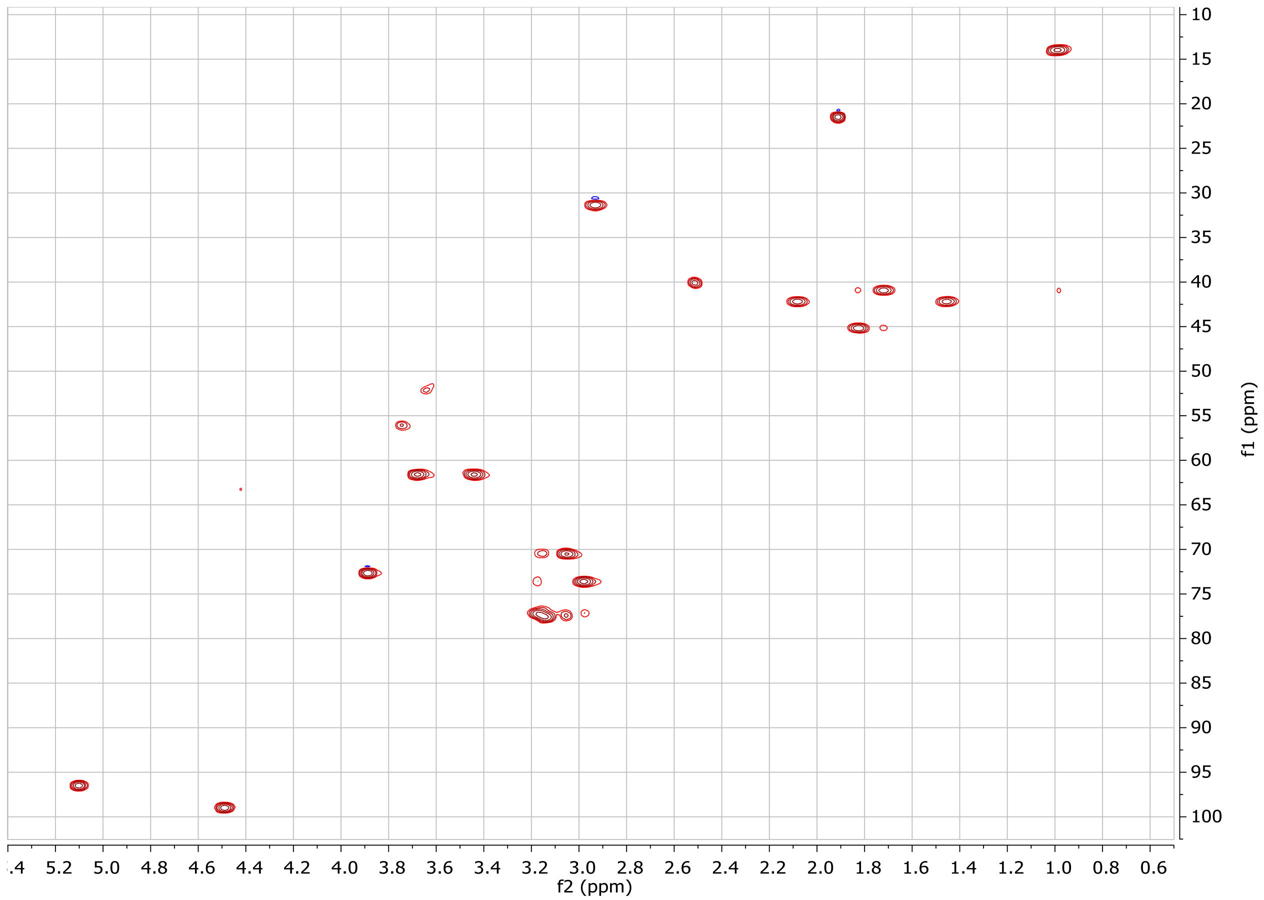

HMBC of loganic acid:

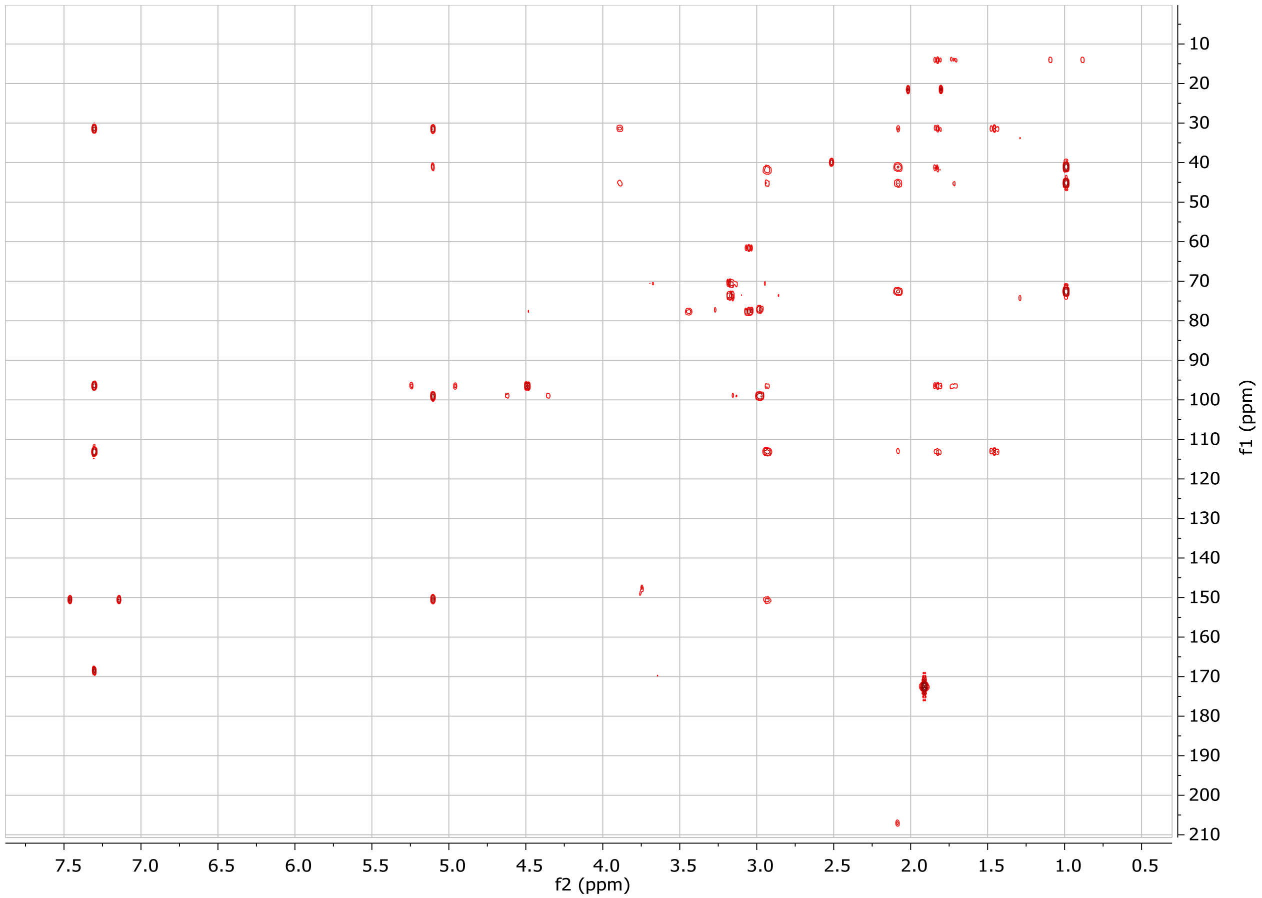

ROESY spectrum of loganic acid:

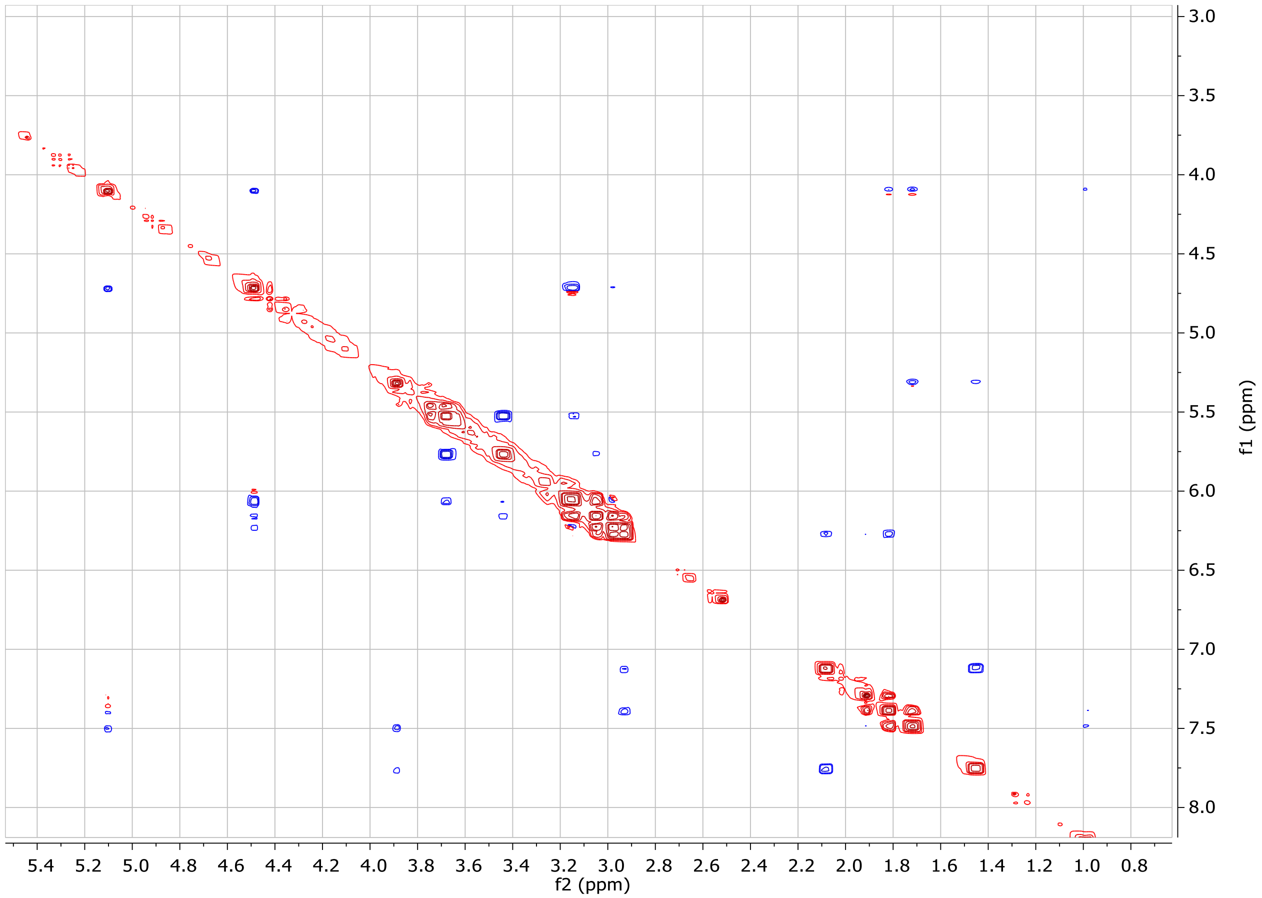

5.4 5.2 5.0 4.8 4.6 4.4 4.2 4.0 3.8 3.6 3.4 3.2 3.0 2.8 2.6 2.4 2.2 2.0 1.8 1.6 1.4 1.2 1.0 0.8
f2 (ppm)
3.0 3.5 4.0 4.5 5.0 5.5 6.0 6.5 7.0 7.5 8.0
f1 (ppm)
