# Supplementary material for: Application of Cornelian Cherry Iridoid-Polyphenolic Fraction and Loganic Acid to Reduce Intraocular Pressure
Source: Evid Based Complement Alternat Med. 2015 Jun 1;2015:939402. doi: 10.1155/2015/939402 (PMC4466386; doi:10.1155/2015/939402)
Supplement: Supplementary file 1 [file 939402.f1.zip › 939402supp2Suplementary data Fig.1s . HPLC Chromatogram of Loganic acid 245nm i 520nm (1).docx]

LA

245 nm

520 nm

mAU

**Supplementary materials, Figure 1s.** HPLC-DAD chromatograms (254 nm; 520 nm) of main compound of loganic acid fraction from the cornelian cherries (*Cornus mas* L.) fruits.
