# Supplementary material for: Application of Cornelian Cherry Iridoid-Polyphenolic Fraction and Loganic Acid to Reduce Intraocular Pressure
Source: Evid Based Complement Alternat Med. 2015 Jun 1;2015:939402. doi: 10.1155/2015/939402 (PMC4466386; doi:10.1155/2015/939402)
Supplement: Supplementary file 1 [file 939402.f1.zip › 939402supp3Suplementary data Fig.2s . HPLC Chromatogram of iridoid-polyph fraction 245nm i 520nm_21 April (1).docx]

LA

mAU

520 nm

245 nm

Pg gal

Cy rob

Cy gal

Pg rob

Df gal

Co

Q-g

Kf-g

5-CQA

3-CQA

**Supplementary materials, Figure 2s.** HPLC-DAD chromatograms (254 nm; 520 nm) of main compounds of iridoid-polyphenolic fraction from the cornelian cherries (*Cornus mas* L.) fruits.

3-CQA: 3-O-caffeoylquinic acid; LA-loganic acid; 5-CQA: 5-O-caffeoylquinic acid; Df gal: 3-O-galactoside; Cy gal: cyanidin 3-O-galactoside; Cy rob: cyanidin 3-O-robinobioside; Pg gal: pelargonidin 3-O-galactoside; Pg rob: pelargonidin 3-O-robinobioside; Q-g: quercetin 3-O-glucuronide; Kf-g: kaempferol 3-O-galactoside; Co: cornuside
